# Frnakenstein: multiple target inverse RNA folding

**DOI:** 10.1186/1471-2105-13-260

**Published:** 2012-10-09

**Authors:** Rune B Lyngsø, James WJ Anderson, Elena Sizikova, Amarendra Badugu, Tomas Hyland, Jotun Hein

**Affiliations:** 1 Department of Statistics, University of Oxford, Oxford OX1 3TG, UK; 2 Department of Computer Science, University of Oxford, Oxford OX1 3QD, UK; 3 ETH Zürich, Department of Biosystems Science and Engineering, 4058 Basel, Switzerland; 4 Mathematics Institute, University of Oxford, Oxford OX1 3LB, UK

**Keywords:** RNA, Inverse folding, Genetic algorithm, Riboswitch

## Abstract

**Background:**

RNA secondary structure prediction, or folding, is a classic problem in bioinformatics: given a sequence of nucleotides, the aim is to predict the base pairs formed in its three dimensional conformation. The inverse problem of designing a sequence folding into a particular target structure has only more recently received notable interest. With a growing appreciation and understanding of the functional and structural properties of RNA motifs, and a growing interest in utilising biomolecules in nano-scale designs, the interest in the inverse RNA folding problem is bound to increase. However, whereas the RNA folding problem from an algorithmic viewpoint has an elegant and efficient solution, the inverse RNA folding problem appears to be hard.

**Results:**

In this paper we present a genetic algorithm approach to solve the inverse folding problem. The main aims of the development was to address the hitherto mostly ignored extension of solving the inverse folding problem, the multi-target inverse folding problem, while simultaneously designing a method with superior performance when measured on the quality of designed sequences. The genetic algorithm has been implemented as a Python program called Frnakenstein. It was benchmarked against four existing methods and several data sets totalling 769 real and predicted single structure targets, and on 292 two structure targets. It performed as well as or better at finding sequences which folded *in silico* into the target structure than all existing methods, without the heavy bias towards CG base pairs that was observed for all other top performing methods. On the two structure targets it also performed well, generating a perfect design for about 80% of the targets.

**Conclusions:**

Our method illustrates that successful designs for the inverse RNA folding problem does not necessarily have to rely on heavy biases in base pair and unpaired base distributions. The design problem seems to become more difficult on larger structures when the target structures are real structures, while no deterioration was observed for predicted structures. Design for two structure targets is considerably more difficult, but far from impossible, demonstrating the feasibility of automated design of artificial riboswitches. The Python implementation is available at http://www.stats.ox.ac.uk/research/genome/software/frnakenstein.

## Background

The function of the RNA molecule depends on the way it folds – structural changes can change protein binding sites, or affect activity for ribozymes, for example. RNA folding allows the single strand of nucleotides to fold upon itself and form more complex structures such as helical junctions and pseudoknots; almost as soon as RNA started to be sequenced, methods were established to determine the structure from the sequence of nucleotides. Early attempts include [1], who simply summed over all possible secondary structures and evaluated them with respect to free-energy functions. Biological and thermodynamical principles have since been applied to formulate more advanced free-energy functions facilitating more accurate and efficient predictions, which have been used to great effect in methods such as UNAFold [2] and RNAfold [3]. Stochastic Context-Free Grammars have also been used to great effect in programs such as Pfold [4,5]. For a review of RNA secondary structure prediction, see [6] or [7].

The inverse RNA folding problem is defined as follows: given a particular RNA secondary structure (target structure), find a sequence of base pairs that would fold into this structure. One could adopt two possible solution techniques: either find an exact match, i.e. a sequence whose predicted structure matches the target structure exactly, or look for a sequence whose predicted structure is as close as possible to the target structure (a suboptimal solution). Then, the inverse folding problem becomes an optimization problem: the goal is to minimize the distance metric defined between a given target structure and the predicted structure of a sequence. Here we consider only base pairs {*A*−*U*,*U*−*A*,*C*−*G*,*G*−*C*,*G*−*U*,*U*−*G*}, and, since experimental verification of the folded structure is not feasible, the structure predicted computationally by RNAfold is used as a proxy for the true secondary structure of a designed sequence.

There are several existing approaches to the RNA Inverse Folding Problem. RNAInverse [3] is the most basic method, inspired by local guided search. Given a target structure, it produces a random sequence that is then randomly changed at points where its predicted structure differs from the target structure. RNA-SSD [8], INFO-RNA [9], NUPACK: Design [10], and Inv [11] are also local guided search based methods, using various combinations of intelligent initial sequence design and/or hierarchical decomposition of the target structure. MODENA [12,13] is the only algorithm that introduces a genetic algorithm approach. This approach is multi-objective: it aims to maximise the closeness to the target structure and minimize the free energy of a solution. It facilitates a better exploration of the search space, and avoids the limited prediction capabilities of the local guided search methods. However, a strong focus on energy minimisation causes an extreme bias towards C-G base pairs.

However, there are currently no implementations available capable of solving the inverse folding problem under multiple structural constraints. To our knowledge, the only existing method for inverse folding with multiple structure targets was published by [14]. This method has not been made publically available as part of the Vienna package. With the current interest in using bio-molecules in nano-technology, the ability to design artificial riboswitches reacting to changes in conditions will become increasingly important. Hence an implementation capable of solving the inverse folding problem for multiple structures is a key development in structure design.

## Methods

The inverse folding is implemented by a fairly standard genetic algorithm (GA) approach [15], expanding a population by mutations and recombinations and selecting the most fit individuals for propagation to the next generation. The main deviation from a completely generic GA is that the method is aware of the aim of designing sequences folding into one or more target structures. Rather than search the full sequence space, we direct the search by ensuring all sequences can fold into the target structure(s) forming only canonical base pairs. This can be viewed as a similar approach to the local search/adaptive walk on a hierarchical decomposition of the target structure implemented by some methods [8,10,11,16], except that the recombination operation chooses a random decomposition and assesses two complementary structural components in conjunction, rather than independently.

In addition to a random search, our method also implements several strategies for more directed evolution. Instead of making uninformed evolutionary changes and leave it to the selection part of the GA to direct the search, it is also possible to bias the choice of change towards e.g. mutating positions with a predicted structure that does not match the target, or in a recombination towards parts that have a good match to the target structure in the regions where they contribute sequence. These strategies are all available through command line options (and as classes when the implementation is used as a library rather than stand-alone program) to allow experimentation and tailoring to specific applications. Through experimentation, a set of parameters was found which worked reasonably on all types of structure, which is set as a pre-defined default. In the following, we will use $T={\left\{\tau \right\}}_{\tau \in T}$ to denote a target consisting of a non-empty set of structures, all of length *n*, and *s* to denote a sequence in the GA population.

### Positional fitness

A key concept in the GA is the fitness of positions. This allows directing evolutionary changes by choosing unfit positions for mutations – an approach also taken in [8,10] and to a lesser extent in [3,9,11] – and regions with fit positions for recombination. Positional fitnesses take a value of 1 for maximally unfit positions and a value of 0 for maximally fit positions.

We define positional fitness schemes relative to a single target structure *τ*. If T consists of multiple target structures, the final positional fitness is computed as the average positional fitness over all structures in T. Let *τi* denote the required structure for position *i* in *τ*, i.e. either forming a base pair with another position *j* or unpaired. Let *σ*denote the optimum structure predicted for *s* (i.e. the minimum free energy structure), and let S[i] denote the set of possible structures for position *i*, i.e. base pairs with position *i* as one base in the pair and the unpaired configuration. Finally, let p:S[i]↦R be the marginal Boltzmann probability in the form of a mapping from positional structure to marginal probability. Our method provides the following choices for the fitness of position *i*, denoted *f*[*i*].

***Scheme 1.*** Binary indicator of whether position has correct predicted structure, *f*[*i*]= 1 −* δ*_*σ* [*i*],*τ*[*i*]_ where *δ *is the Kronecker delta. This corresponds to the *μ*(*sτ*) based objective in [10], providing a binary view of whether the design matches at position *i* that may be too coarse grained. However, it does also capture *σ*is correct at position *i*, or the design needs to change.

***Scheme 2.*** Boltzmann probability of target structure, *f*[*i*] = 1 − *p*(*τi*). This corresponds to the *n*(*sτ*) objective in [10], providing a measure of how likely a structure drawn from the Boltzmann ensemble is to match *τ*at position *i*. This provides more fine grained information than scheme 1, but measures proximity rather than match to *τ*.

***Scheme 3.*** Truncated negative logarithm of Boltzmann probability of target structure, *f*[*i*] = min{−log(*p*(*τi*))/100,1}. This penalises decreasing probability to match *τ*with increasingly severity, and is particularly useful for multiple structure targets where the average corresponds to a sum of logarithms, thus penalising designs failing to match just one of the target structures. Truncation is performed to allow mapping to [0,1].

***Scheme 4.*** Binary indicator of whether probability of target structure exceeds threshold $\theta ,f\left[i\right]=1_{p\left(\tau ,\left[i\right]\right)<\theta }$. This allows requiring all elements of *τ*to be present with a given probability when sampling the Boltzmann ensemble, but being oblivious to further improvements beyond that. It is particularly useful when T specifies a multi-stable design, i.e. a target with multiple structures for the same temperature, by measuring the number of target structures the design matches with probability at least *θ *at position *i*.

***Scheme 5.*** Sigmoid transformed difference between Boltzmann probability of target structure and most probable alternative structure, $f[i]=\frac{1}{2}+\frac{3}{4}x-\frac{1}{4}x^{3}$ where $x=\underset{\nu \in S[i]\setminus \{\tau [i]\}}{\max}\{p(\nu )\}-p(\tau [i])$. This allows accepting lower probabilities of *τi*in regions where the structure is generally less well defined, although a position is still most fit iff *p*(*τi*) = 1. The transformation corresponds to $\int_{-1}^{x}-(y+1)(y-1)\text{dy}$, normalised to yield values in [0,1], and causes changes to have larger effects when the probability of *τi*and the most probable alternative are close to being equal.

The *p* and *σ* are computed at the temperature specified for *τ*, and may thus differ between the *τ*’s when a multiple structure target is specified.

Finally, the following positional fitness schemes specifically designed for multiple structure targets T are defined.

***Scheme 6.*** Minimum Boltzmann probability of target structures, $f[i]=1-\underset{\tau \in T}{\min}\{p(\tau [i])\}$.

***Scheme 7.*** Product of Boltzmann probabilities of target structure, $f[i]=1-\underset{\tau \in T}{\prod }p(\tau [i])$.

For single structure targets, they are equivalent to the Boltzmann scheme, scheme 2. For multiple structure targets, scheme 6 exclusively focuses on the worst fitness over all target structures, while scheme 7 includes Boltzmann probabilities from all target structures. However, by multiplying the probabilities, having a low fitness on just a single target structure will have a much more notable effect, than under the sum implicit in the averaging of scheme 2.

In addition to a single of the above schemes, there is the possibility of using a weighted combination of any subset of them to define positional fitness. E.g. combining the first two schemes would divide positions based on whether they have a predicted structure matching the target, but further graduate the fitness by the marginal Boltzmann probability of the target structure at each position.

The concept of positional fitness underpins most operations of the GA: mutation, recombination, selection, and termination. Whenever fitness of a region (for recombination cross over point selection) or the entire sequence (for selection and termination) is needed, this is obtained as the sum of the positional fitnesses in the region or sequence. Different positional fitness schemes can be used for these four aspects, with the limitation that negative logarithms of Boltzmann probabilities are only used for mutation, and product of Boltzmann probabilities cannot be used for mutation.

### Fitness and objective

Often fitness and objective of GAs are considered equivalent, but we make the distinction of using fitness for the selection in each round of the GA and objective for determining when an adequate solution has been found and the search can be terminated. In a standard design problem where the aim is to find a sequence folding to one specific target structure, it is natural to base the objective on whether positions are correct in the predicted structure and terminate when the number of errors reaches 0. However, a more fine grained selection may be desirable, for example substituting or combining the number of errors with scheme 2 – instead of choosing randomly between two sequences with e.g. 10% positions that are wrong in the predicted structure, we would prefer the one with higher probabilities of positions being correct.

A global, i.e. non-positional, scheme

***Scheme 8.*** Logarithm of structure probabilities in Boltzmann ensemble and their variance: $f=\bar{x}+\xi \left(\frac{1}{\left|T\right|}\underset{\tau \in T}{\sum }{x_{\tau }}^{2}-{\bar{x}}^{2}\right)$ where $\bar{x}=\frac{1}{\left|T\right|}\underset{\tau \in T}{\sum }x_{\tau }$ and *x*_*τ *_= −log*p*_*τ *_is the negative logarithm of the probability of target structure *τ *in the Boltzmann ensemble of sequence *s*, and *ξ* ≥ 0 is the weight assigned to the contribution from the variance

based on the cost functions discussed in [14] and corresponding to the *Π*(*sτ*) objective in [10], is also available for defining fitness and objective. This provides a means of requiring an exact match to the target structures, rather than basing the fitness on the distance to predicted or expected structure as in e.g. schemes 1 and 2. This is particularly relevant when designing for multiple structures at the same or very similar temperatures, where some of the position based schemes may be confused by designs where positions exhibit a good match to varying subsets of the target structures.

Finally, to maintain diversity in the GA population, the fitness can be augmented with a weighted contribution from the average Hamming distance to already selected sequences. If P denotes the set of sequences already selected in the selection stage at the end of a generation in the GA, each remaining candidate sequence *s* has its fitness augmented by $\zeta \underset{t\in P}{\sum }(n-h(s,t))/|P|$, where *h*(*s*,*t*) is the Hamming distance between sequences *s* and *t*, and *ζ* > 0 is the weight assigned to the contribution from diversity, before selecting the next individual carried forward.

### Mutation

The position targeted for mutation in a sequence is chosen either uniformly at random, or with probability proportional to positional fitnesses. Similarly, sequences can be chosen for mutation either equally many times, uniformly at random, or with probability proportional to the reciprocal of the sequence fitness (with sequences with fitness 0 given twice the probability of the otherwise most fit sequences).

When choosing a new nucleotide for a position chosen for mutation, we want to maintain *compatibility* with all target structures. That is, the modified sequence should fold into each target structure using only canonical Watson-Crick and GU wobble base pairs. When the target consists of a single structure, unpaired positions can be updated independent of the rest of the sequence, while base pairing positions can be updated by sampling a new base pair for the two positions independent of the rest of the sequence. However, with two or more target structures, dependencies can extend to more positions, as a position can be base paired to multiple other positions in the different target structures.

The *target dependency graph* (TDG) (denoted *dependency graph* in [14]) implied by T is the graph on nodes {1,…,*n*} where two nodes *i, j* are connected by an edge iff ∃τ∈T:i·j∈τ, where *i* · *j*denotes a base pairing of positions *i* and *j*. The compatibility of a position will depend, directly or indirectly, on all positions in the connected component it belongs to in the TDG, so the entire connected component may have to be updated in a mutation. It can be observed from ( [14], Theorems 1 & 2), that with canonical base pairing compatible sequences will exist iff the TDG is bipartite, and if |T|≤2 the TDG will be bipartite. If |T|≥3, no compatible sequence may exist. For example, if three target structures contain base pairs *i*·*j*, *j*·*k*, and *i*·*k*, respectively, then there is no assignment of nucleotides to positions *i, j*, and *k* that will leave all base pairs canonical.

In [14] formulas for sampling an assignment of nucleotides on a connected component when the maximum degree of any node is at most 2 is provided. However, we have chosen a simpler, heuristic update algorithm for two reasons. First, our method was developed to also cope with larger sets of target structures. Secondly, even for |T|=3 the maximum node degree may be 3 and hence one may suspect assignment of nucleotides, i.e. colouring of the nodes, uniformly at random to be difficult – with a three letter nucleotide alphabet with base pairs allowed between any two non-identical nucleotides, the problem becomes *#***P** hard [17]. Finally, it is unclear whether sampling uniformly from the set of compatible assignments is the best strategy. As G’s and U’s can pair with two other types of nucleotides, while C’s and A’s can pair with only one other type, the set of compatible assignments will be biased towards a high GU content.

The following forms our method for sampling nucleotides on a connected component in the TDG, starting with position *i*, ensuring all positions form canonical base pairs following the update. 

 Choose *σ*from {A,C,G,U}∖{*s*[*i*]} and set *s*[*i*] =* σ*

*F *= {*i*}, *N *= {*j*∣*i* · *j*}

**while***N* ≠ * ∅ ***do**

 Choose *j* ∈ *N *uniformly at random

 Choose *σ *from $\cap_{k\in F:j\cdot k}C(s[k])$ and set *s*[*j*] =* σ*

*F *=* F*∪{*j*}, *N *=* N*∪{*k*∣*j*·*k*}∖*F*

end while

where *j* · *k *denotes that two nodes are connected by an edge in the TDG and C(σ) is the set of nucleotides compatible with *σ*. It performs a traversal of the connected component of *i*, at each step choosing a random node neighbouring the already updated nodes. New nucleotides are drawn from a distribution which can be specified – if the default uniform distribution is used, this will tend to favour high GU content for the same reason as for choosing complete compatible assignments uniformly at random discussed above – truncated to the possibilities allowed. If the current nucleotide is among the choices, there is an option either always to keep the current nucleotide (to limit collateral effects of a mutation) or to bias the draw with 1 − *f* [*i*] for the current nucleotide and *f* [*i*] for alternatives (to allow the current positional fitness to affect the probability of a change).

### Recombination

Due to the hierarchical nature of RNA secondary structures, the GA uses recombination mimicking gene conversion rather than cross over, i.e. an infix of one sequence is recombined with the corresponding prefix and suffix of the other sequence. The easiest way to keep all base pairs canonical, is to always take two positions forming a base pair in a target structure from the same sequence. If we create a recombinant on sequences *s* and *t* from a crossover point *i*,*j *by forming the sequence *s*[1*..i*] *t* [*i* + 1*..j*] *s* [*j* + 1*..n*], no base pairing positions come from different sequences iff there are no base pairs in the target structure(s) for which one of *i* + 1 and *j* is inside the base pair and the other one is outside the base pair. This means that we can partition break points into sets of *pairwise permissible points*, such that the aim of taking base pairing positions from the same sequence is achieved iff crossover points are chosen as pairs of points from the same set in this partition. For single structure targets, these sets are exactly the loops of the structure, when stacking base pairs are viewed as internal loops of size 0, and the set of all external positions. The following outlines the procedure used for constructing the sets of pairwise permissible points, where sets of size 1 are discarded. The target is denoted by T, and when the algorithm terminates *C* is the set of non-singleton sets of points that are pairwise permissible. 

*C*={{0,…,*n*}}

**for**τ∈T,i·j∈τ**do**

*C*^*′ *^=* ∅*

 for *S* ∈ *C* **do**

*C*^*′ *^=* C*^*′*^∪

 (*X* ∈ {* S*∩[*i*, *j*)[ ,*S*∩ ([0,*i*[∪[*j*,*n*])}:|*X*|>1

endfor

*C *=* C*^*′*^

end for

As a starting point, pairs of points are chosen by first choosing a set of pairwise permissible points with probability proportional to the set size, then choosing a pair from the set uniformly at random, ensuring an overall uniform probability that a point is chosen. This distribution can be biased proportional to

$$\phi_{i,j}^{s,t}=\overset{n}{\underset{k=1}{\sum }}\left\{\begin{array}{l} f_{s}[k]\;\text{if}\;k\leq i\;\text{or}\;k>j\\ f_{t}[k]\;\text{if}\;i<k\leq j \end{array}\right)$$

where *f*_*s*_ and *f*_*t*_ are positional fitnesses for *s* and *t* respectively. Similarly, the pair of sequences *s, t* can be chosen uniformly at random, based on individual fitness as described for mutations above, or based on the sum over all pairs *i, j* of permissible points of ${\left(\phi_{i,j}^{s,t}\right)}^{2}$ to preferentially choose pairs of sequences complementing each other.

### Initialisation

Initialisation of sequences in the starting population can either be done randomly, by sampling nucleotides for each connected component in the TDG as outlined for mutation, but without the presence of current nucleotides, or by running RNAinverse from a random starting point. The latter option, an approach also used by RNAexinv [18], allows solutions to be found rapidly for easier targets. When RNAinverse is used and the target T consists of multiple structures, a random τ∈T is chosen for each run. Hence, the initial sequences may not be compatible with T. Additionally there is also the option to read the initial sequences from file, for example if specific sequence motifs are present it may be desirable to litter the initial population with them – it does also provide a simple means for using an alternative inverse folding method to create the initial population of sequences.

### Data

Data was taken from two main sources, to benchmark Frnakenstein and other inverse folding methods. The first data set used in our benchmarks is the data set used in [12]. This was downloaded from the MODENA website. It consists of a structure from each of the 29 out of the first 30 families in Rfam [19], with the tmRNA family (RF00023) left out due to a high content of pseudoknot forming base pairs. We refer to this data set throughtout as the *Rfam* data set.

Secondly, data was taken from RNASTRAND [20], which itself takes data from many sources [21-25]. The data was filtered so that the sequences and structures could ensure reliability of predictions. We removed identical sequences and disregarded synthetic data and sequences with ambiguous base pairs. Further, any sequences with greater than 80% base pair similarity with another structure in the data set were removed, as well as all sequences with pseudoknots, as RNAfold does not predict pseudoknots. The resulting data set consisted of 397 RNA molecules, containing 363 unique secondary structures with a total length of 55,025, which we refer to as the *RNASTRAND* data set.

However, with both data sets, it may be possible that there is no sequence which RNAfold will fold into the reference structure, and so the method might not be able to acheive 100% accuracy, due to RNAfold, not the search heuristic. Consequently the sequences corresponding to the structures in the RNASTRAND data set were re-folded using RNAfold, so there is known to be at least one sequence which will correctly fold. This dataset will be denoted as the *RNASTRAND-Refolded*, and consists of 383 unique structures with a total length of 56,606.

## Results and discussion

### Multi-structure targets

**Figure 1:**
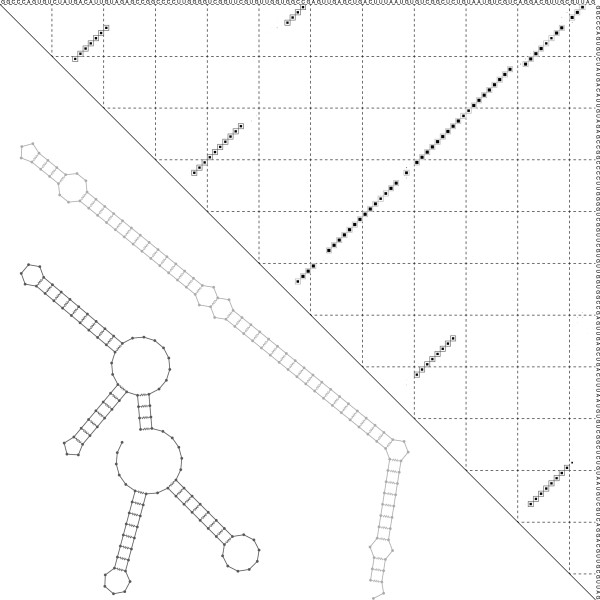
**Design of artificial SV11 RNA.** Dot plot of base pair Boltzmann probabilities for the designed sequence for the bistable SV11 target. Superimposed on the dot plot is a plot of base pairs in the two metastable SV11 structures, shown with open squares in different shades of grey. The secondary structures are also shown in the same shades of grey. Dots reflecting Boltzmann probabilities were rescaled by a factor of 0.75 to clearly separate them from any enclosing square representing a structure base pairs. The two conformations, that share no base pairs, are also show:, the native state (top) and meta-stable state (bottom).

One of the main objectives of Frnakenstein was to develop a method capable of solving the inverse folding problem under multiple structural constraints. As mentioned earlier, the only existing method for inverse folding with multiple structure targets was published by [14]. This method has not been made part of the Vienna package, so we were unable to benchmark against this method. However, the paper does provide an example of a 115 nucleotide RNA molecule, SV11, that exists in two major conformations, a meta-stable multicomponent structure and a rod-like native state. They present a design for these two structures as target in ( [14], Figure seven). Applying Frnakenstein to this two-structure target we obtained the design shown in Figure 1. This provides an almost perfect match to the target, including the isolated base pair 33·79 completely missing in ( [14], Figure seven) – as observed in [9,16], designing sequences for targets with isolated base pairs is at best difficult and sometimes impossible.

For all benchmarks on multiple structure targets we set the number of generations to the total target length, i.e. the number of structures in the target multiplied by the length of the sequence to be designed. Again Frnakenstein was run with default values, which means that compared to the single structure target default outlined above, positions for mutations were chosen based on a 1:1:1 combination of schemes 1, 2, and 3; cross over points were chosen based on a 1:1:2 combination of schemes 1, 2, and 7; fitness was based on a 1:1:2:4 combination of schemes 1, 2, and 7, and the diversity maintaining contribution from Hamming distances, except for the SV11 example above where a fitness based on scheme 8 with *ξ *= 1 and an objective based on scheme 4 with *θ *= 1/3 was used.

To some extent the SV11 target poses an impossible challenge, as we cannot find a sequence having both conformations as the most stable structure. Hence, for bi-stable targets, we cannot measure performance by simply reporting successes and failures. To avoid this problem, we decided to test the performance of multiple structure target design by providing target structures at two different temperatures. We folded the 304 sequences with at most 200 nucleotides of the RNASTRAND data set under 20°C and 37°C, simulating a change from room temperature to normal body temperature. After eliminating duplicates, 291 two structure targets remained.

177 targets, with a total of 11,188 nucleotides, had identical structures at the two temperatures. 114 targets, with a total of 10,578 nucleotides, had different structures at the two temperatures, with an average of 16.9 positions where the structures differed. Even when the structures differ, it will be possible to design a sequence that successfully folds to the correct target structure at each temperature, allowing a simple and easy to understand measure on performance of number of successes. This does not directly test the ability to design a bi-stable molecule. However, it does test performance on multiple structure targets in a related realistic scenario for inverse RNA folding, where the aim is to design a molecule that under different conditions, either remains stable or performs as a riboswitch reacting to the change in conditions.

Table 1 shows the performance of Frnakenstein on this data set. Results are categorised based on whether target consisted of identical or different structures, and whether the best design was successful for both, one, or none of the target structures. A success is Frnakenstein finding a sequence which folds correctly into the target structure. For each category we list the number of targets in the category (*n*), the average length of the targets ( $\bar{l}$), and where relevant the average number of differences between the targets ( $\bar{d}$) and the average number of differences between target and design per target structure the design failed on ( $\bar{\Delta }$). Note that identical structures mean that a sequence has to fold into the same structure at both 20°C and 37°C.

**Table 1:** Performance on two structure targets

	**Both**			**One**				**None**			
	***n***	$\bar{l}$	$\bar{d}$	***n***	$\bar{l}$	$\bar{d}$	$\bar{\Delta }$	***n***	$\bar{l}$	$\bar{d}$	$\bar{\Delta }$
Identical structures	173	61.5	–	0	–	–	–	4	139.0	–	3.3
Different structures	54	71.5	7.5	32	94.1	19.9	8.1	28	132.5	31.8	9.6

Performance on 291 two-structure targets generated by folding shorter RNASTRAND at 20°C and 37°C.

Successful designs were obtained for 227 targets. Of the remaining 64 targets, the design folded correctly at one temperature for 32 targets, with an average of 8.1 positions where the predicted structure of the design differed from the target at the other temperature. For the remaining 32 targets, the design did not fold correctly at either temperature, with an average of 8.8 positions being wrong. It should be remembered that targets were created by folding a specific sequence at two different temperatures using RNAfold, so in all cases we know that a perfect design does exist. Comparing this to the results obtained on RNASTRAND-Refolded, it is evident that the multiple structure target problem is considerably more difficult than the single structure target problem, in particular when the target structures differ.

### Single-target structures

Since Frnakenstein works for single targets too, the performance of our method on single targets could be benchmarked against other methods that are publicly distributed as source code or executables. This includes RNAinverse [3], MODENA [12], INFO-RNA [9], NUPACK: Design [10], and Inv [11]. All benchmarks were done on a 48 core Dell PowerEdge with 2.3GHz AMD Opteron processors and 128GB 1.3GHz memory. Despite the availability of source code of RNA-SSD [8,16], it could not be successfully compiled, so the web server was used where appropriate.

For each method being benchmarked, efforts were made to give it the same number of attempts at the problem, despite them employing different search heuristics. Our method and MODENA were both run 10 times with a population size of 50, and a number of generations equal to the number of positions in the structure (with a minimum of 50 generations). RNAinverse, RNA-SSD, INFO-RNA, NUPACK: Design, and Inv were all run 500 times with default parameters. All methods apply the Vienna RNA package for structure prediction, except for NUPACK: Design that uses the NUPACK suite, and Inv, which uses its own thermodynamic model. NUPACK allows interior loops of arbitrary sizes, whereas the Vienna package limits interior loops to a maximum size of 30. This allows NUPACK: Design to report a successful design on target structures with large interior loops, where all other methods will necessarily fail due to this limitation of RNAfold. Notably this is seen on the RF00016 and RF00024 structures in the Rfam data set, which contains interior loops with 83 and 67 unpaired nucleotides, respectively. Inv is even more restrictive on permissible structures, allowing only structures with a minimum stack size of 3, and minimum arc length of 4. This means that many trusted structures in, for instance, the Rfam database it deems as invalid, and thus will produce an error, suggesting it will perform badly in the benchmarks.

Frnakenstein was run with default values, which means mutation was biased towards fit sequences, positions were chosen based on a 2:1 combination of schemes 1 and 2; recombination was biased towards pairs of sequences with good complementary match and cross over points chosen based on a 1:1 combination of schemes 1 and 2; fitness was based on a 1:1:2 combination of schemes 1 and 2, and the diversity maintaining contribution from Hamming distance to already selected sequences, while objective was simply the number of erroneous positions in the predicted structure, cf. scheme 1. As one cannot determine statistically what fitness, mutation, and recombination schemes will optimise success of the algorithm, the defaults were determined heuristically to make Frnakenstein most likely to find a successful sequence for the target structure.

#### Rfam structures

Results from benchmarking on the Rfam data set are shown in Table 2. Accession numbers have in the table been shortened by replacing the three leading 0s with ·. A ‘success’ indicates that the program has found a sequence which folds *in silico* into the target structure, with RNAfold being used by all excepting NUPACK: Design, which uses its own thermodynamic folding model similar to RNAfold. Each entry then lists the fraction of runs that successfully designed a sequence for the target, with non-zero fractions in bold, followed by the average running time in seconds. RNA-SSD results were obtained from the RNA-SSD server, with run times as reported from the server – run times are only reported for successful runs, so the average run time listed is the average over successful runs. An asterisk for Inv indicates that Inv reported that the target structure it was given was invalid and so did not make an attempt. Several of the NUPACK: Design benchmarks could not be fully completed, as each of the 500 independent searches were still incomplete after several days of run time, having neither found a successful design nor terminated the search.

**Table 2:** Performance on Rfam data set

**Acc.**	**Len.**	**Frnakenstein**	**MODENA**	**RNA-SSD**	**INFO-RNA**	**RNAinverse**	**NUPACK**	**Inv**
01	117	**1.0** / 1204	**1.0** / 17	**0.01** / 8.2	**0.95** / 1.2	**0.01** / 61	**0.29** / 11613	∗
02	151	**1.0** / 13505	**1.0** / 19	0.0 / –	0.0 / 175	0.0 / 277	0.0 / 141999	∗
03	161	**0.1** / 9997	**1.0** / 37	0.0 / –	**0.01** / 112	0.0 / 304	0.0 / 50698	∗
04	193	**1.0** / 198	**1.0** / 27	0.0 / –	**0.27** / 64	**0.10** / 164	**1.0** / 5597	∗
05	74	**1.0** / 0.14	**10.** / 4.8	**1.0** / 0.34	**0.99** / 0.31	**0.87** / 1.4	**1.0** / 148	**0.29** / 9.2
06	89	**1.0** / 27	**1.0** / 5.9	**0.98** / 3.4	**0.66** / 4.6	**0.06** / 20	**0.99** / 299	∗
07	154	**1.0** / 115	**1.0** / 22	**0.05** / 5.1	**0.85** / 7.4	**0.08** / 70	**0.95** / 1611	∗
08	54	**1.0** / 0.09	**1.0** / 2.8	**0.96** / 0.05	**1.0** / 0.15	**0.95** / 0.22	**1.0** / 47	**0.22** / 1.1
09	348	**1.0** / 129057	**1.0** / 123	0.0 / –	0.0 / 4127	**0.01** / 7100	**0.78** / 111487	∗
10	357	0.0 / 245868	0.0 / 180	0.0 / –	0.0 / 4046	0.0 / 8007	0.0 / 92211	∗
11	382	0.0 / 500078	0.0 / 184	0.0 / –	0.0 / 7040	0.0 / 16634	0.0 / 77273	∗
12	215	**1.0** / 5455	**1.0** / 35	0.0 / –	**0.01** / 329	**0.01** / 558	**0.98** / 2825	∗
13	185	**1.0** / 65	**1.0** / 27	0.0 / –	**0.37** / 61	**0.09** / 127	**1.0** / 190	∗
14	87	**1.0** / 0.15	**1.0** / 7.3	**0.94** / 0.09	**1.0** / 0.30	**1.0** / 0.27	**1.0** / 34	∗
15	140	**1.0** / 333	**1.0** / 13	0.0 / –	**0.51** / 29	**0.05** / 118	**1.0** / 40696	∗
16	129	0.0 / 18734	0.0 / 11	0.0 / –	0.0 / 102	0.0 / 124	**0.48** / 10167	∗
17	301	**1.0** / 318	**1.0** / 117	0.0 / –	**0.94** / 21	**0.23** / 263	**1.0** / 703	∗
18	360	**1.0** / 210591	**1.0** / 180	0.0 / –	**0.01** / 4260	**0.01** / 5305	0.0 / 101125	∗
19	83	**1.0** / 1.0	**1.0** / 6.3	**0.4** / 0.63	**0.98** / 0.52	**0.57** / 3.7	**1.0** / 46	∗
20	119	0.0 / 3149	0.0 / 10	0.0 / –	0.0 / 7.8	0.0 / 15	0.0 / 810	∗
21	118	**1.0** / 0.23	**1.0** / 13	**0.99** / 0.44	**1.0** / 0.77	**0.96** / 1.7	**1.0** / 130	∗
22	148	**1.0** / 293	**1.0** / 16	0.0 / –	**0.15** / 46	**0.02** / 97	**1.0** / 5335	∗
24	451	0.0 / 138348	0.0/ 182	0.0 / –	0.0 / 1530	0.0 / 4170	**0.23** / 8533	∗
25	210	**1.0** / 11838	**1.0** / 29	0.0 / –	**0.06** / 132	0.0 / 463	**1.0** / 10420	∗
26	102	**1.0** / 290	**1.0** / 6.1	0.0 / –	**0.04** / 50	**0.02** / 60	**1.0** / 1429	∗
27	79	**1.0** / 0.23	**1.0** / 8.1	**1.0** / 0.29	**1.0** / 0.49	**0.82** / 1.2	**1.0** / 112	**0.86** / 9.7
28	344	**0.3** / 197498	0.0 / 125	0.0 / –	**0.01** / 4627	0.0 / 6003	**0.09** / 31920	∗
29	73	**1.0** / 33	**1.0** / 4.1	**0.82** / 1.4	**0.67** / 0.58	**0.2** / 1.5	**0.72** / 229	∗
30	340	**1.0** / 133627	**1.0** / 115	0.0 / –	**0.01** / 1896	0.0 / 5011	0.0 / 118373	∗
Total successes		24	23	10	22	19	22	3

Comparison of five inverse folding methods on 29 Rfam structures.

This data set was sufficiently small that RNA-SSD could be included in the benchmark by manually uploading the targets to the RNA-SSD server. Our method and MODENA, the only two genetic algorithm based methods in the benchmark, exhibits the best performance, each successfully designing sequences for 23 of the 29 targets. INFO-RNA also performs well, with 21 successful designs, while RNA-SSD and RNAinverse have more limited success, and Inv performing poorly. Every target Inv considered to not be invalid it succeeds with, but it is so limited on what it permits that it does not attempt the majority of structures.

Of the 5 target structures for which all RNAfold based method failed, two (RF00016 and RF00024) have internal loops with more than 30 nucleotides, which makes it impossible to reach a successful design as discussed above. All methods, including NUPACK: Design, failed on the remaining three (RF00010, RF00011, and RF00020). These all contain a bulge of a single nucleotide separated from either a large hairpin loop or the exterior of the structure by an isolated base pair. With the current energy parameters, it is impossible to design a sequence where the same structure with the isolated base pair removed would not be more stable [16]. Hence, Frnakenstein is the only method which does design sequences for all targets which are possible. For the remaining targets, the best designs differ in one or at most a few base pairs, e.g. by introducing an isolated base pair separating the large internal loops into two parts.

If we look at how nucleotides and base pairs are utilised by the different methods, Table 3 shows the distribution of base pairs and nucleotides in the successful designs for each method. The first group shows the distribution over the three types of base pairs in paired positions in the targets, the second group shows the nucleotide distribution for unpaired positions in the targets, and the last group shows the overall nucleotide distribution in the sequences. MODENA, INFO-RNA, and to an extent NUPACK: Design clearly differs from the rest of the methods, by having a base pair distribution heavily biased towards GC base pairs. This should come as little surprise. INFO-RNA starts from a sequence designed to have lowest possible free energy over all sequences when folding in to the target structure. MODENA uses the free energy on the target structure as one of the objectives it optimises. This emphasis on minimising the free energy on the target structure causes a heavy bias towards the more stable CG base pairs with INFO-RNA likely to start from a sequence with almost exclusively CG base pairs and MODENA consistently preferring replacing other base pairs with CG base pairs. What causes the slightly less pronounced bias for NUPACK: Design is less clear, though. Observing that MODENA furthermore has a strong bias towards A in unpaired positions, the method is close to limiting itself to designing sequences over a one letter alphabet for unpaired positions and a two letter alphabet for paired positions. If sequences are designed to plant in simulated data, e.g. to test an RNA gene finder, it will also be preferable to obtain sequences with more natural distributions.

**Table 3:** Nucleotide distribution in designed sequences

	**Paired**			**Unpaired**				**Total**			
	**GC**	**AU**	**GU**	**A**	**C**	**G**	**U**	**A**	**C**	**G**	**U**
Original data	0.57	0.30	0.13	0.30	0.20	0.23	0.27	0.23	0.24	0.28	0.24
Frnakenstein	0.55	0.36	0.09	0.32	0.31	0.09	0.29	0.25	0.29	0.19	0.26
MODENA	0.82	0.18	0	0.82	0.06	0.06	0.06	0.48	0.22	0.22	0.07
INFO-RNA	0.93	0.06	0.01	0.36	0.22	0.20	0.22	0.19	0.35	0.32	0.14
RNA-SSD	0.56	0.44	0	0.32	0.24	0.19	0.25	0.27	0.26	0.24	0.23
RNAInverse	0.46	0.41	0.14	0.29	0.25	0.21	0.25	0.23	0.24	0.26	0.26
NUPACK	0.73	0.27	0	0.42	0.26	0.09	0.22	0.28	0.31	0.22	0.18
Inv	0.32	0.39	0.28	0.30	0.26	0.22	0.22	0.20	0.21	0.30	0.29

Comparison of the nucleotide distributions of the successfully designed sequences from different methods on the Rfam dataset, with distribution observed across the original sequences from Rfam shown in the first row.

The other four methods have less bias in the base pair uses, although only Frnakenstein, RNAinverse, and Inv utilise wobble GU base pairs to any real degree. Indeed, the base pair distribution observed in the Frnakenstein distribution is very close to the distribution observed in the original data. There is, in the unpaired nucleotides, a mild overrepresentation of Cs and a mild under representation of Gs, though. This is perhaps due to the increased thermodynamic stability in CG base pairs– it is perhaps easy to have Cs unpaired, or Gs unpaired, but too many will form a pair eventually. RNAInverse, on the other hand, had an unpaired distribution very close to the real data set, but the base pair distribution is off a little. While it may in many cases be less important whether the distributions observed in the designed sequences are heavily biased, one consequence of this bias will be a reduced diversity in the set of solutions that are generated. In this sense, given Frnakenstein’s performance against RNA Inverse and Inv, when application dictates a sensible nucleotide distribution, Frnakenstein is the clear winner.

Frnakenstein was designed with little focus on running time, choosing Python as implementation language for the ease of development and flexibility it offers. Additionally, the more advanced choices in mutation and recombination selection provide additional computational burden. Not only do you now have to calculate the full partition function for the thermodynamic model necessary to obtain Boltzman probabilities, but the selection of individuals for recombination and the recombination points themselves becomes considerably more computationally expensive. It is thus not overly surprising that among the methods tested Frnakenstein is one of the slowest. Only NUPACK: Design vies with Frnakenstein for bottom slot regarding speed. Even accounting for the fact that average running times should be divided by success ratio to get an approximate value of total time until first successful design, Frnakenstein and NUPACK: Design tend to be three to four orders of magnitude slower than the other four tested methods on some targets. For easy targets, Frnakenstein mitigates this concern by the application of RNAinverse for sequence initialisation. For applications where minimising time to find a successful design is the key priority, as opposed to nucleotide distribution, MODENA is a strong contender with run time at most a few minutes and a high rate of success on most of the Rfam targets.

**Figure 2:**
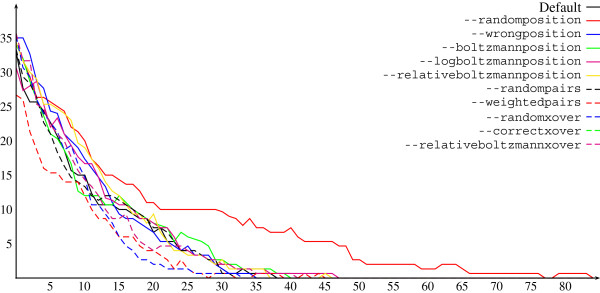
**Analysis of positional fitness schemes.** Plot showing the minimum objective value in the population through the generations of the GA for the default parameters (solid black line) and ten variations where a single feature is changed by invoking the respective options shown in the legend. These corresponds to choosing positions for mutation uniformly at random, as well as based on positional fitness schemes 1, 2, 3, and 5; choosing pairs of sequences for recombination uniformly at random or based on individual fitnesses; and choosing recombination points uniformly at random, as well as based on positional fitness schemes 1 and 2.

Additionally, we analysed the performance of different positional fitness schemes. A subset of structures were taken from the Rfam data set and Frankenstein run many times with different positional fitness options, and for each run, the minimum objective recorded for each generation. For each positional fitness option, averages were then taken over runs, and results are found in Figure 2. The main thing which is clear from this is the much slower reduction in minimum objective seen when the mutations are decided uniformly at random. This is the method employed by MODENA. Otherwise, it is difficult to distinguish the different positional fitness schemes. The default has been chosen for good success ratio (in our experience), but other choices may be faster/better for specific situations.

#### RNAStrand data

Results for both the RNASTRAND and RNASTRAND-Refolded can be found in Table 4. A ‘success’ indicates that the program has found a sequence which folds *in silico* into the target structure, with RNAfold being used by all excepting NUPACK: Design, which uses its own thermodynamic folding model similar to RNAfold. The data sets contain 363 and 383 structures, respectively. The numbers reported are the number of target structures for which a sequence was successfully designed. Again, several of the NUPACK: Design benchmarks for the RNASTRAND data set could not be fully completed, as each of the 500 independent searches were still incomplete after several days of run time, having neither found a successful design nor terminated the search.

**Table 4:** Performance on RNASTRAND data set

	**Frnakenstein**	**MODENA**	**INFO-RNA**	**RNAinverse**	**NUPACK**	**Inv Frnakenstein**
RNASTRAND	189	178	196	176	185	73
RNASTRAND-Refolded	383	377	383	336	383	113

Successes of the benchmarked approaches on the RNASTRAND and RNASTRAND-Refolded.

These results confirm the general picture seen for the Rfam data set: Frnakenstein, MODENA, and INFO-RNA have similar success rates with RNAinverse lagging slightly behind, although only on the re-folded structures and not quite to the same degree as for the Rfam data set. Inv once again performs poorly, although there are more structures it permits in this data set, once again succeeding on all of them. The dependency of performance on target length, cf. Table 5, is somewhat inconclusive, with all methods except Inv performing best, by far, on the bin with length between 118 and 151 nucleotides. Given the limited size of the bins, strong conclusions should probably not be drawn from this. However, whereas all methods do seem to struggle more with longer targets, there does seem to be a tendency for RNAinverse, and to a lesser extent MODENA, to show a more rapid decline in performance on long targets. On shorter targets, these two methods perform as well as the other methods. For the re-folded targets, Frnakenstein, INFO-RNA, and NUPACK: Design do achieve a 100% success rate, with successful designs for all targets across the full range of target lengths, so performance is more affected by the nature of the target than target length. The biases in base pair and nucleotide distributions for the successful designs were similar to the ones observed for the Rfam data set (data not shown), as should be expected.

**Table 5:** Length dependency of success rate

**Range**	**10-23**	**24-36**	**37-56**	**57-77**	**78-98**	**99-117**	**118-151**	**152-269**	**270-311**	**312-1037**
**Av. length**	**17.9**	**29.4**	**46.2**	**69.8**	**86.7**	**108.0**	**127.7**	**205.6**	**293.8**	**528.0**
**Bin size**	**38**	**34**	**36**	**41**	**34**	**36**	**35**	**36**	**36**	**37**
Frnakenstein	0.50	0.50	0.56	0.76	0.62	0.44	0.97	0.47	0.17	0.22
MODENA	0.47	0.50	0.56	0.76	0.59	0.44	0.97	0.44	0.03	0.14
INFO-RNA	0.50	0.50	0.56	0.78	0.65	0.53	0.91	0.50	0.28	0.19
RNAinverse	0.50	0.50	0.56	0.78	0.62	0.42	0.97	0.42	0.08	0.00
NUPACK	0.50	0.50	0.56	0.78	0.56	0.44	0.97	0.47	0.14	0.16
Inv	0.44	0.38	0.47	0.51	0.15	0	0	0	0	0

The 363 unique structures of the RNASTRAND data set were binned according to length in 10 bins of roughly equal size, and for each bin, the range of lengths covered by the bin, the average length of structures in the bin, the number of structures in the bin are listed, as well as the success ratio on each bin computed for each method.

## Conclusions

In this paper we have described how to use a genetic algorithm approach to find useful solutions for the inverse RNA folding problem. The method allows a combination of the predicted minimum free energy structure and the computed Boltzmann distribution over the ensemble of structures to be used to guide the main aspects of the genetic algorithm, in particular mutation, recombination, and selection. It performed as well, or better, than the other methods tested on all benchmarks, without introducing strong biases in the composition of the designed sequences.

One of the major advantages of our method is that it allows multiple structures, either at identical or different conditions, to be specified as targets. To our knowledge, only one previously published method has this capability, and the software implementing this method is only available on request. While the benchmarks were done on two targets, there are no upper restrictions to how many targets can be aimed for.

Our method uses the RNA secondary structure prediction software as a black box. While a more efficient solution could be obtained by a more complex interaction with the folding software, allowing reuse of already computed values when mutating and recombining sequences, the chosen approach makes Frnakenstein much more flexible. The folding method can be replaced with relative ease, e.g. to use a grammar based method or a method capable of predicting structures with pseudoknots, simply by providing an alternative implementation of the module invoking and parsing the output from the folding software. Combining predictions from several folding methods, possibly using a multi-objective framework similar to MODENA, allows designs more robust to the uncertainties of structure prediction, and is an interesting direction for future research.

## Competing interests

The authors declare that they have no competing interests.

## Authors’ contributions

RL and JWJA developed the initial idea for the method, which was tested and implemented by RL, AB, TH, and ES. Benchmarks were designed and carried out by JWJA, RL, ES, AB, and TH. Manuscript was drafted by RL, JWJA, and ES. All authors read and approved the final manuscript.
